# Berberine Ameliorates Inflammation in Acute Lung Injury via NF-κB/Nlrp3 Signaling Pathway

**DOI:** 10.3389/fnut.2022.851255

**Published:** 2022-02-25

**Authors:** Jiyu Chen, Yanli Huang, Xiaohong Bian, Yan He

**Affiliations:** ^1^Clinical Trials Center, The Affiliated Hospital of Guizhou Medical University, Guiyang, China; ^2^Office of Academic Research, The Affiliated Hospital of Guizhou Medical University, Guiyang, China; ^3^School of Life Science and Technology, China Pharmaceutical University, Nanjing, China

**Keywords:** berberine, ALI, Nlrp3, NF-κB, inflammation

## Abstract

The inflammatory response is the key pathophysiological character of acute lung injury (ALI). Berberine (BBR), a natural quaternary ammonium alkaloid, plays a functional role in anti-inflammation both *in vitro* and *in vivo*. However, the underlying mechanism between BBR and ALI has not been expounded. Here, we found that BBR improved the permeability of pulmonary and repressed the inflammatory factors in the lipopolysaccharides (LPSs)-induced ALI model. We demonstrated that BBR could suppress the expression of phosphorylated nuclear factor-kappa B (NF-κB) and further restrain the downstream gene nucleotide-binding domain and leucine-rich repeat protein-3 (Nlrp3). Moreover, we also revealed that BBR could directly interact with Nlrp3 protein. After knocked down of Nlrp3 by using siRNA, the protective role of BBR was abrogated *in vitro*. The expression of IL-1β and IL-18 was downregulated by BBR *via* the two signaling pathways. Notably, in Nlrp3 deficient mice, the protective effect of BBR was abolished. These findings demonstrate that BBR has a depressant effect on inflammatory response caused by LPS *via* regulating NF-κB/Nlrp3 signaling pathway, providing a potential therapeutic strategy in ALI.

## Introduction

Acute lung injury (ALI) and further worse form, acute respiratory distress syndrome (ARDS), are the major cause of acute respiratory failure, even death (1). The development of ALI is linked with the disorder of inflammation and damage of alveolar barriers (2). Notably, nearly 75,000 people were dead causing by ALI every year in America (3). Numerous progress has been performed to understand the underlying pathophysiology, nevertheless, only a little treatment reduces the death leading by ALI (4). Hence, it is urgent to discover an efficient therapeutic strategy for ALI.

Lipopolysaccharides (LPSs), found on the outer membrane of Gram-negative bacteria, were widely applied to establish the ALI model of different animals (5–9). The inflammasome, a cytosolic surveillance, is active by the activation of systemic inflammation and the damage of human pulmonary microvascular endothelial cells (HPMECs) induced by LPS revive (10). The activated inflammasome, named nucleotide-binding domain and leucine-rich repeat (NLP) protein-3 (Nlrp3), further promotes the maturation of inflammatory cytokines interleukin-1β (IL-1β) and IL-18 (11). Hence, eliminating excessive inflammation is a therapeutic strategy for ALI.

Berberine (BBR), as an inhibitor of inflammation, plays a key treatment role in various diseases. It is widely used to treat diarrhea and gastroenteritis in China since the 1950s (12). Additionally, published research has demonstrated that BBR also possesses a therapeutic effect on diabetes mellitus rats against cognitive decline, aggravating insulin resistance, and promoting the repair of canalicular laceration by suppressing the inflammation response (13–15). However, the underlying mechanism of BBR in ALI caused by LPS is unclear.

In this study, we revealed that BBR attenuates inflammation response by regulating nuclear factor-kappa B (NF-κB)/Nlrp3 signaling pathway in response to ALI caused by LPS. The phosphorylation of NF-κB and downstream gene Nlrp3 are suppressed by treatment with BBR, downregulating the accumulation of IL-1β and IL-18 induced by LPS. Overall, our results demonstrate that BBR, a natural production, could ameliorate inflammation and may serve as a therapeutic drug for ALI.

## Methods and Materials

### Animal Models

The animal experimental protocol was performed under the supervision of the Animal Experimental Ethics Committee of Guizhou Medical University (No. 2101452). The 8-week-old male C57BL/6 mice were purchased from Cavens Experimental Animal Company (Changzhou, Jiangsu) and Nlrp3^−/−^ mice were obtained from GemPharmatech Company (Nanjing, China). The mice were divided into four groups: the phosphate buffered saline (PBS)/0.5% DMSO group, the PBS/BBR group, the LPS/0.5% DMSO group, and the LPS/BBR group. The ALI animal models were performed with LPS induction as previously (16). The mice were injected BBR (10 mg/kg; Sigma-Aldrich) into the peritoneal cavity first 24 h and again at 2 h before LPS injection. Then, the mice were collected after LPS (15 mg/kg; Sigma-Aldrich) incubation for 8 h. Notably, the LPS was administrated intraperitoneally.

### Histological Analysis

After collection, the lung tissues were fixed in 4% paraformaldehyde (PFA) (Servicebio) overnight. The lungs were cut into 4 μm thickness and performed to H&E staining. The analysis of lung injury was measured as previously described (17). Briefly, there were five scales: 0 = no injury; 1 = up to 25% injury of the field; 2 = up to 50% injury of the field; 3 = up to 75% injury of the field; and 4 = diffused injury.

### Cell Culture

Immortalized HPMECs were purchased from Chemical Book (Guangzhou). The cells were cultured in Dulbecco's modified eagle medium (DMEM) medium supplemented with 10% foetal bovine serum (FBS), 100 units/ml penicillin and 100 μg/ml of streptomycin at 37°C with 5% CO_2_. For the detection of mRNA and protein levels, samples were collected for further analysis after stimulation with LPS (100 ng/ml) and treatment with BBR (2.5 μM) for 48 h.

### Bronchoalveolar Lavage Fluid Collection and Analysis

The BALF collection was performed as previously (18). The BALF was collected by cleaning the pulmonary with a tracheal cannula with 1 ml precooled PBS, which was repeated three times. The BALF was centrifuged at 400G for 10 min to collect the cells. Then, the protein concentration of the supernatant was detected by using bicinchoninic acid (BCA) protein assay kit (Yeasen), and the count of cells was measured by calculating instrument (BodBoge; JSY-SC-021H).

### Evaluation of Lung Permeability

The detection of lung permeability was performed as previously described (19). Evans blue (20 mg/kg; Sigma-Aldrich) was injected into mice through the caudal vein for circulation 1 h before collection. The lung tissues were washed through right ventricular puncture with saline for 5 min. The lung tissues were weighed after drying at 56°C for 2 days and the dye was extracted in 1 ml of formamide (Sigma-Aldrich) for 24 h at 60°C. In addition, the content of Evans blue was detected at 620 nm.

### Evaluation of HPMECs Permeability

The detection of cells permeability was performed as previously described (20). Briefly, HPMECs were seeded in 0.4-μm transwell inserts and stimulated with LPS or treatment with BBR. After that, FITC-dextran (1 mg/ml; 40 kDa; Thermo Fisher Scientific) was added to the upper for incubating 30 min. Then, 50 μl of medium obtained from the bottom chamber was detected fluorescence at 520 nm.

### Determination of Reactive Oxygen Species (ROS)

The content of ROS was detected as previously described (21). The frozen lung sections were treated with dihydroethidium (DHE) dye (Beyotime, China) at 37°C for 30 min, then using PBS washed for 5 min for three times. In addition, the HPMECs were fixed with 4% PFA (Electron Microscopy Sciences) for 15 min, and then incubated with DHE for 30 min at 37°C and then washed three times of PBS for 5 min. A confocal scanning microscope was used to detect the ROS level and further analyzed by Image J software.

### Enzyme-Linked Immunosorbent Assay

The levels of IL-6 (SEKM-0007 and SEKH-0013, Solarbio), tumor necrosis factor-α (TNF-α) (SEKM-0034 and SEKH-0047, Solarbio), IL-10 (SEKM-0010 and SEKH-0018, Solarbio), IL-18 (SEKH-0028 and SEKM-0019), and IL-1β (SEKM-0002 and SEKH-0002) in lung tissues and cell cultures were measured according to the instructions.

### Western Blot

The Western blot was performed as in a previous study (22). The lung tissues and cells were collected and added proteinase inhibitor (Vazyme), phosphatase inhibitor (Vazyme), and radio immunoprecipitation assay (RIPA) (Beyotime). Then the mixes were split by using the grinding machine (Jingxin, Shanghai; JXFSTPRP-CL) and centrifuged at 12,000 rpm for 15 min to collect the supernatant. The concentration of supernatant protein was detected by using BCA assay kit (Yeasen). After separation and transfer, the corresponding proteins were measured by using the following antibodies: Nlrp3 (Abcam; ab263899), Phospho-NF-κB (Affinity; AF2006), and Actin (Proteintech; 66009-1-Ig).

### Quantitative Reverse Transcription-PCR (qRT-PCR)

The qRT-PCR was carried out as previously described (23). Total RNA from lung tissues and cells were isolated using the TRIzol Reagent (Vazyme). The concentration and quality of messager RNA (mRNA) were assessed on NanoDrop 2000 (Thermo Fisher Scientific). After the synthesis of cDNA was performed using HiScript III 1st Strand cDNA Synthesis Kit (Vazyme) and T100 Thermal Cycler (Bio-Rad). Then, qRT-PCR was performed using ChamQ SYBR Color qPCR Master Mix (Vazyme) and QuantStudio 5 (Thermo Fisher). Primers sequences were shown as follows:

IL-6 (Human) forward: ATGTAGCCGCCCCACACAGC;
        reverse: CATCCATCTTTTTCAGCCAT;
IL-6 (Mouse) forward: TACCACTTCACAAGTCGGAGGC;
        reverse: CTGCAAGTGCATCATCGTTGTTC;
TNF-α (Human) forward:GAGGCCAAGCCCTGGTATG;
        reverse: CGGGCCGATTGATCTCAGC;
TNF-α (Mouse) forward: CACGTCGTAGCAAACCACCAAGTGGA;
        reverse: TGGGAGTAGACAAGGTACAACCC;
IL-10 (Human) forward:TCAAGGCGCATGTGAACTCC;
        reverse: GATGTCAAACTCACTCATGGCT;
IL-10 (Mouse) forward: TCTGTATCACCGAAGCTATGGC;
        reverse: ATGCCGTCCATTGCTTTCAG;
IL-1β (Human) forward: AGGCTGCTCTGGGATTC;
        reverse: GCCACAACAACTGACGC;
IL-1β (Mouse) forward:GCAACTGTTCCTGAACTCAACT
        reverse: ATCTTTTGGGGTCCGTCAACT
IL-18 (Human) forward: TCTTCATTGACCAAGGAAATCGG
        reverse: TCCGGGGTGCATTATCTCTAC
IL-18 (Mouse) forward: GACTCTTGCGTCAACTTCAAGG
        reverse: CAGGCTGTCTTTTGTCAACGA
Caspase 1 (Human) forward: TTTCCGCAAGGTTCGATTTTCA
        reverse: GGCATCTGCGCTCTACCATC
Nlrp3 (Human) forward: AACATTCGGAGATTGTGGTTGGG;
        reverse: GTGCGTGAGATTCTGATTAGTGCTG;
18S forward: GTAACCCGTTGAACCCCATT;
     reverse: CCATCCAATCGGTAGTAGCG;

### Small Interfering RNA Transfection

Small interfering RNAs (siRNAs) were transfected into HPMECs using Lipofectamine™ RNAiMAX Transfection Reagent according to the manufacturer's instructions. siRNAs were obtained from GenePharma (Shanghai). The sequences were as follows.

siNlrp3 sense:GCUGUAACAUUCGGAGAUUGU;
        anti-sense:ACAAUCUCCGAAUGUUACAGC.

### Molecular Docking

The 3D structure of BBR was transformed from 2D version by Molecular Operating Environment (MOE v2019.0102, Chemical Computing Group Inc., Montreal, QC, Canada) through energy minimization. The Nlrp3 protein structure data was downloaded from the protein database (RCSB Protein Data Bank-RCSB PDB, www.pdb.org), and the structure ID was 6NPY. Before docking, the force field of AMBER10: EHT and the implied solvation model of Reaction Field (R-field) were selected. Docking follows the theory of “induced fit,” that is, the conformation of ligand and receptor will change during molecular docking, which is not completely rigid. Small molecule ligands are placed on the active site of the receptor, followed by ligand orientation and conformation search. MOE Dock provides a database of dynamically generated conformations, which are then refined using force field-based methods. The number of docking poses is set to 20. The scoring functions used in this experiment are London dG and generalized born volume integral/weighted surface area (GBVI/WSA) dG scoring functions based on the force field. GBVI/WSA dG, a forcefield-based scoring function, determines the binding free energy (kcal/mol) of the ligand from a given pose. The conformation with the lowest free energy of binding was selected as the most plausible binding mode. Molecular docking result image processing using MOE.

### Statistical Analysis

All the data were expressed as mean ± SD using GraphPad Prism 8. A two-tailed unpaired *t*-test was used to determine the differences between the two groups and two-way ANOVA was employed for comparisons of the differences between multiple groups. Values of *P* < 0.05 were viewed as statistically significant.

## Results

### Berberine Alleviates the Lung Damage Induced by LPS

**Figure 1:**
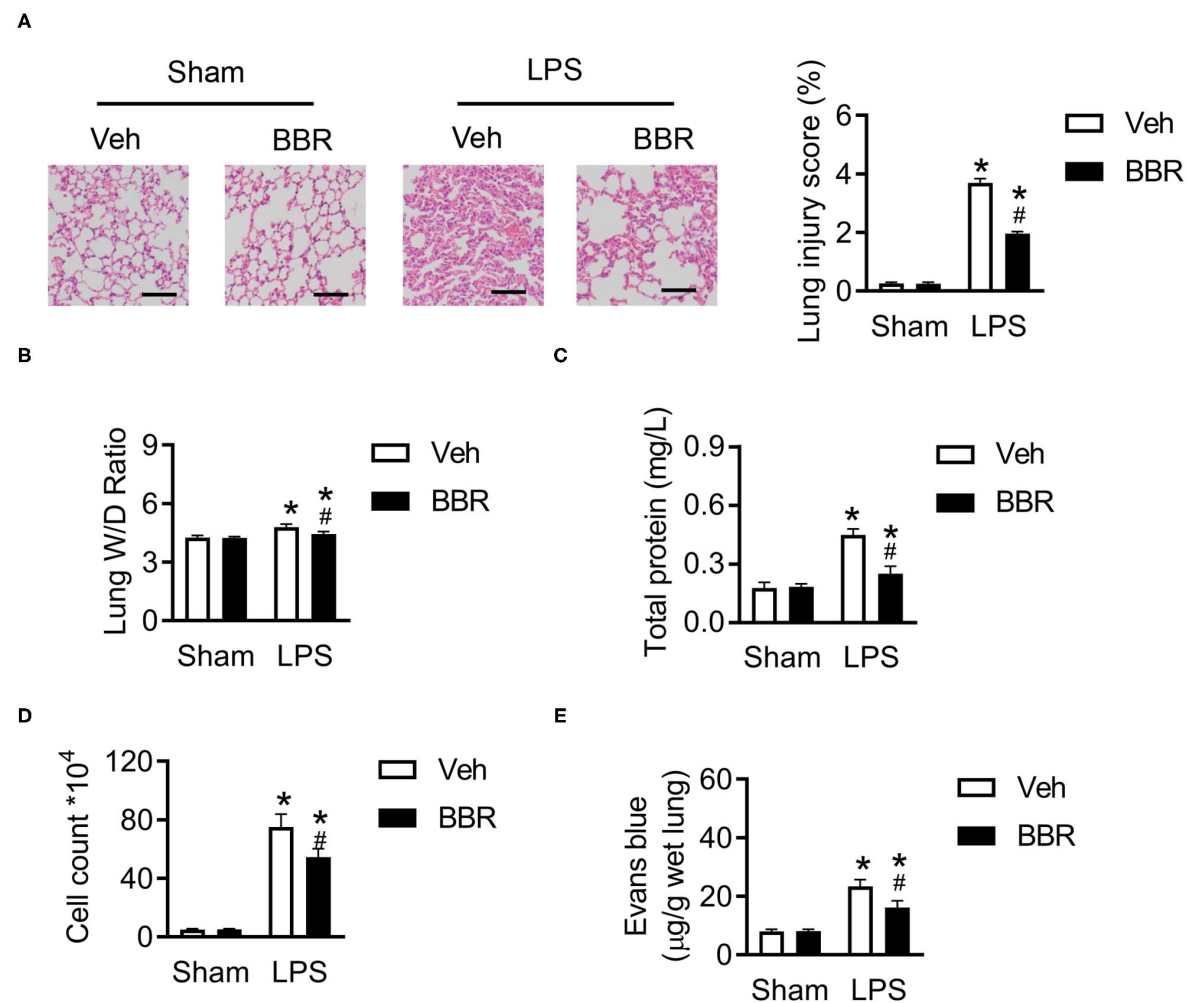
Berberine (BBR) alleviated the permeability and lung tissue injury in C57BL/6 mice with ALI. **(A)** The H&E staining and lung injury score in pulmonary section, *n* = 3, scale bar = 100 μm. **(B)** The ratio of wet lung to dry lung, *n* = 8. **(C,D)** The detection of total protein and cell counts in BALF, *n* = 8. **(E)** The concentration of Evans blue in lung tissue, *n* = 8. Data were expressed as mean ± SD. Statistical significance is indicated as *P < 0.05 vs. the sham + vehicle groups, ^#^*P* < 0.05 vs. the lipopolysaccharide (LPS) + vehicle groups. ALI, acute injury lung; BALF, bronchoalveolar lavage fluid.

To investigate the treatment ability of BBR, we performed a LPS-induced ALI model. The lung from mice after stimulated by LPS for 8 h was collected to perform further analysis. H&E staining of the pulmonary section demonstrated that LPS caused various and severe morphological injuries, including interstitial edema, incomplete morphological structure of pulmonary, infiltration of inflammatory cells, and death of pulmonary endothelial cells, which were improved by treatment with BBR. And the injury scores of the lung were also downregulated by BBR (Figure 1A). Notably, the dysfunction of pulmonary endothelial cells and the incomplete structure of microvascular barriers, as severe pathophysiology in ALI, indicates considerable damage of permeability. Further experimental detection of permeability revealed that the ratio of lung wet to dry weight, contents of total protein, and cell count in BALF were increased significantly induced by LPS, but the trends were partially repressed by BBR treatment (Figures 1B–D). Additionally, the high leaking level of Evans Blue demonstrated that the barriers of pulmonary microvascular endothelial cells were remarkably harmed by LPS, but was rescued by BBR treatment (Figure 1E). Taken together, these results revealed that BBR was capable of ameliorating the injury of pulmonary permeability during ALI.

### Berberine Eliminates Excessive Inflammation in ALI

**Figure 2:**
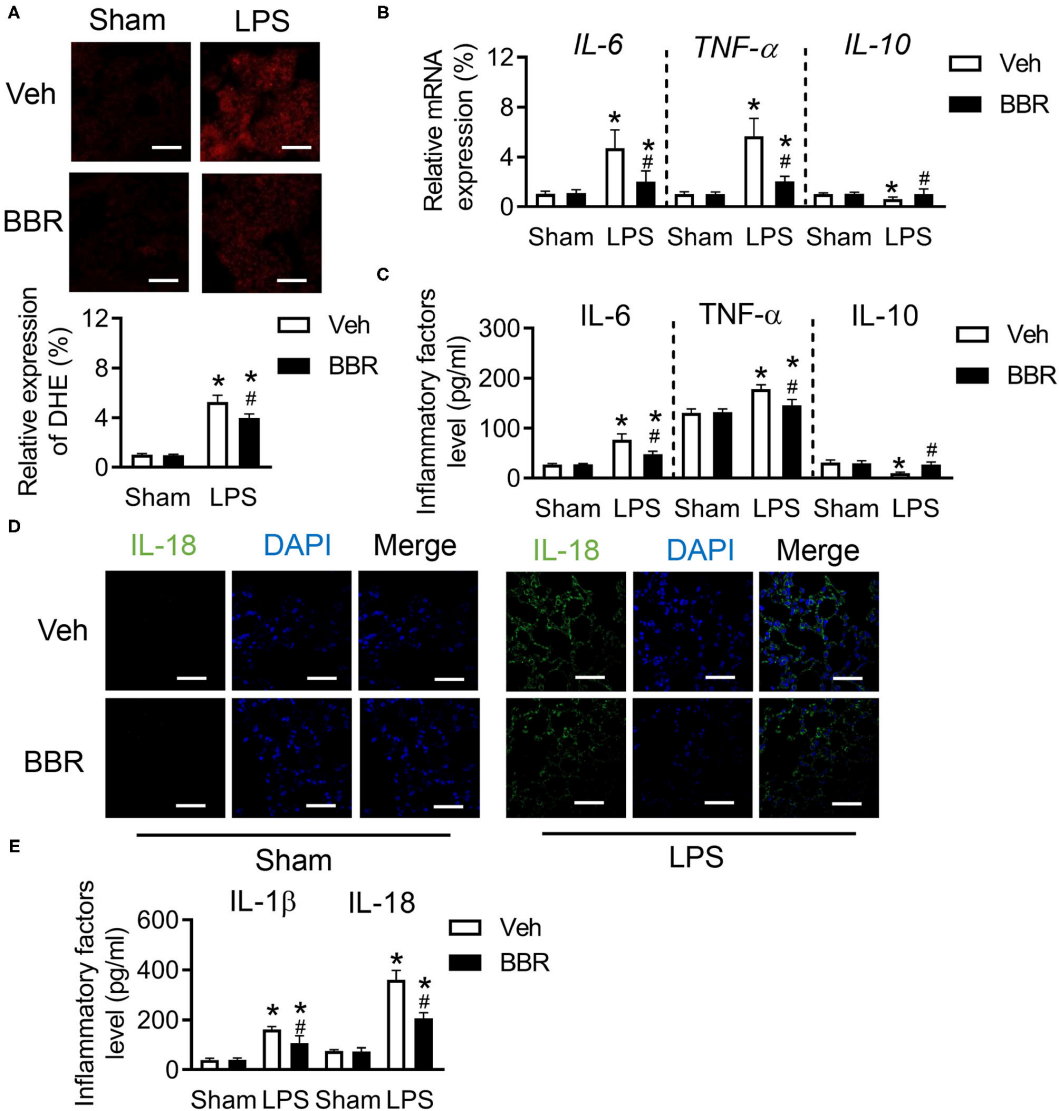
Berberine inhibited the inflammatory cytokines in C57BL/6 mice with ALI. **(A)** The expression of reactive oxygen species (ROS) in pulmonary section and its quantification (down), *n* = 3, scale bar = 20 μm. **(B)** The messager RNA (mRNA) expression of *IL-6, TNF-*α, and *IL-10* in lung tissue, *n* = 8. **(C)** The concentration of IL-6, TNF-α, and IL-10 in lung tissue, *n* = 8. **(D)** The expression of IL-18 in lung section, *n* = 3, bar = 20 μm. **(E)** The concentration of IL-18 and IL-1β in lung tissue, *n* = 8. Data were expressed as mean ± SD. Statistical significance is indicated as *P < 0.05 vs. the sham + vehicle groups, ^#^*P* < 0.05 vs. the LPS + vehicle groups.

The oxidative status consequently was changed during excessive inflammation caused by acute injury (24). We first investigated the level of ROS and found significant upregulation of ROS in the pulmonary section after ALI, which was restrained by BBR treatment (Figure 2A). Notably, the damage of endothelial cells barriers caused infiltration of inflammatory cytokines. We next detected the mRNA expression of *IL-6, TNF-*α, and *IL-10* in lung tissue. Interestingly, the pro-inflammatory factors of *IL-6* and *TNF-*α were remarkably increased on mRNA level after LPS treatment, but BBR decreased their expression (Figure 2B). In contrast, the anti-inflammatory cytokine of *IL-10* was enhanced after BBR treatment in ALI compared to vehicle treatment in ALI (Figure 2B). In addition, the expression tendency of IL-6, TNF-α, and IL-10 in BALF as well as their mRNA expression in lung tissue were also discovered in mice after BBR treatment (Figure 2C). The fluorescence staining and ELISA were used to detect the expression of IL-18 or IL-1β in lung tissues. The results demonstrated that the expression of IL-18 and IL-1β were obviously increased in the LPS-stimulated groups compared with the control groups, and this tendency was substantially reduced by treatment with BBR (Figures 2D,E). In general, BBR might protect the function and structure of the lung tissue by suppressing the infiltration of inflammatory cytokines.

### Berberine Improves the Permeability and Represses the Infiltration of Inflammation in LPS-Stimulated HPMECs

**Figure 3:**
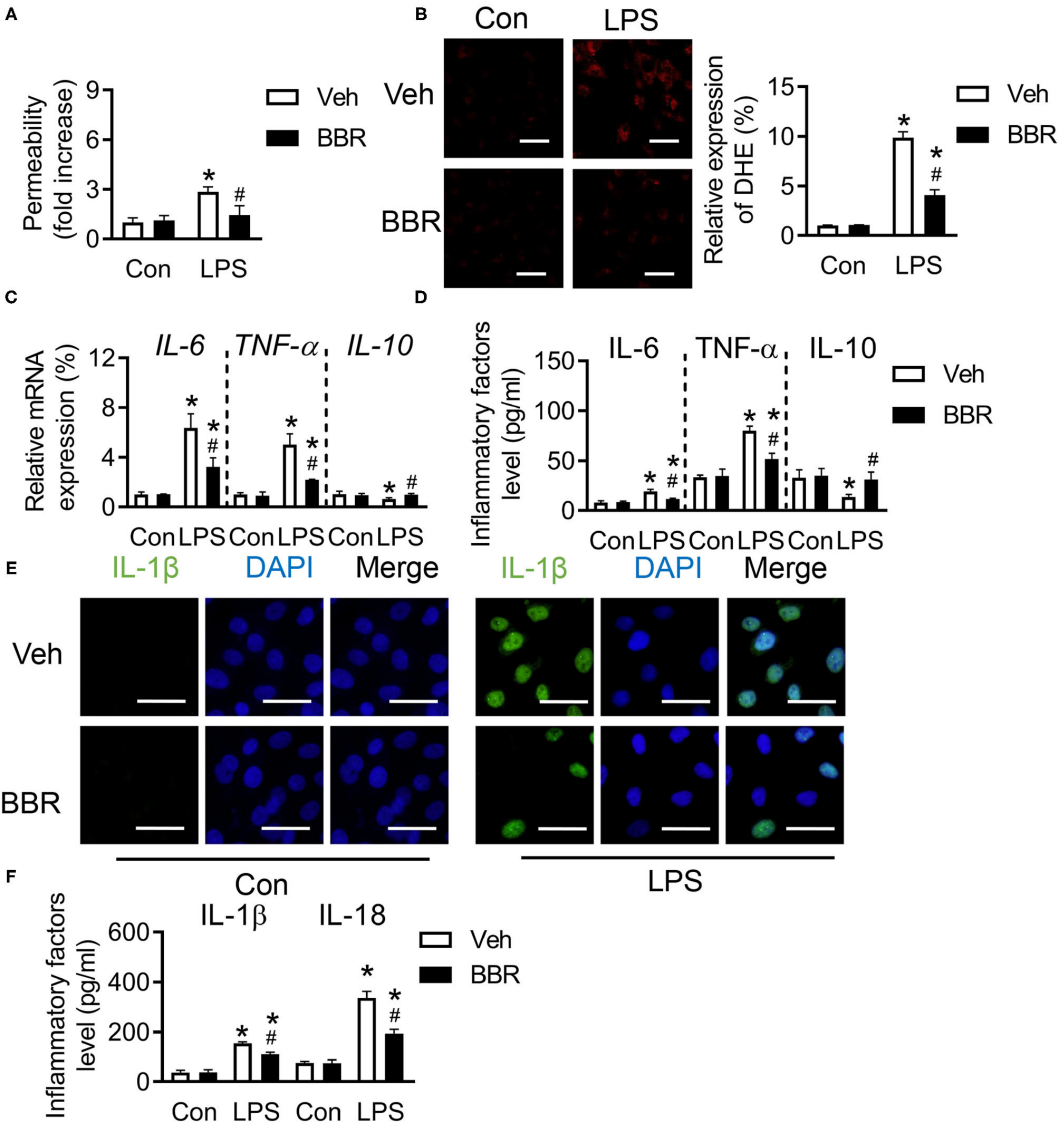
Berberine inhibited the inflammatory cytokines in human pulmonary microvascular endothelial cells (HPMECs) induced with LPS. **(A)** The concentration of Evans blue in HPMECs, *n* = 8. **(B)** The expression of ROS in HPMECs and its quantification, *n* = 3, scale bar = 20 μm. **(C)** The mRNA expression of *IL-6, TNF-*α, and *IL-10* in HPMECs, *n* = 3. **(D)** The concentration of IL-6, TNF-α, and IL-10 in HPMECs, *n* = 4. **(E)** The expression of IL-1β in HPMECs, *n* = 3, bar = 50 μm. **(F)** The concentration of IL-18 and IL-1β in HPMECs, *n* = 4. Data were expressed as mean ± SD. Statistical significance is indicated as *P < 0.05 vs. the sham + vehicle groups, ^#^*P* < 0.05 vs. the LPS + vehicle groups.

To further investigate whether BBR played a protective role in HPMECs, HPMECs were stimulated with LPS after BBR or vehicle treatment to perform ulterior analysis. As expected, the permeability of HPMECs was destroyed by LPS, which would cause more injury. However, this injury was significantly repressed by BBR (Figure 3A). Our results showed the ROS level was restrained by BBR in LPS-induced HPMECs (Figure 3B). Consistent with the animal results, the mRNA expression level and the content of *IL-6* and *TNF-*α in HPMECs were upregulated by LPS, which were both downregulated by BBR (Figures 3C,D). Moreover, the tendency of *IL-10* was also similar to animal experiments (Figures 3C,D). In addition, the fluorescence staining and ELISA were used to detect the expression of IL-1β or IL-18 in HPMECs. The results showed that the expression of IL-1β and IL-18 was upregulated after LPS treatment, which was blocked by treatment with BBR (Figures 3E,F). These results revealed that BBR repressed the infiltration of inflammation *via* protecting the permeability of HPMECs.

### Berberine Attenuates the Expression of Nlrp3 via Regulating the Phosphorylate-NF-κB

**Figure 4:**
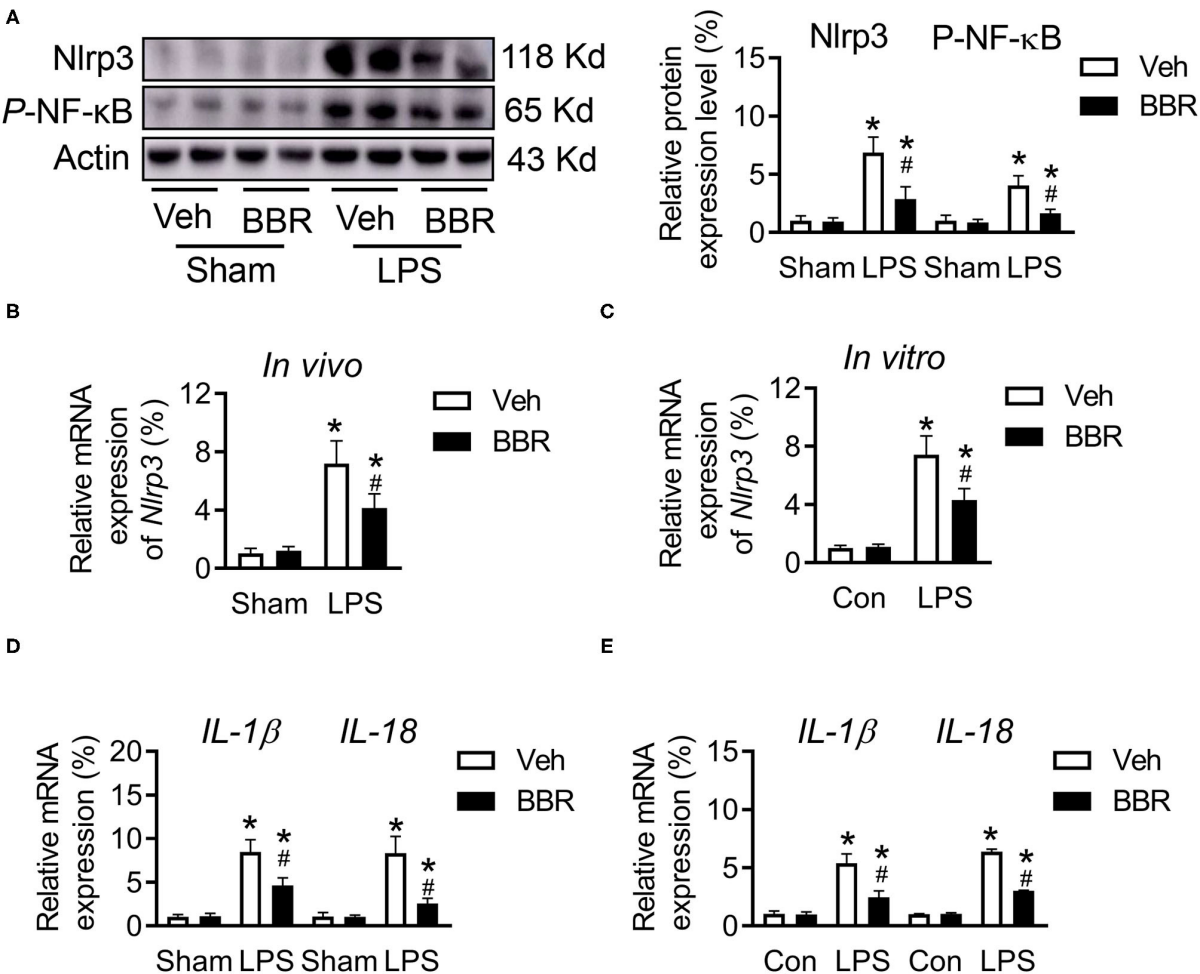
Berberine regulated the expression of phosphorylated NF-κB and Nlrp3 in mice with ALI. **(A)** The expression of Nlrp3 and p-NF-κB at protein level in lung tissue, *n* = 6. **(B)** The mRNA expression of *Nlrp3 in vivo, n* = 8. **(C)** The mRNA expression of *Nlrp3 in vitro, n* = 3. **(D)** The mRNA expression of *IL-1*β and *IL-18* in lung tissue, *n* = 8. **(E)** The mRNA expression of *IL-1*β and *IL-18* in HPMECs, *n* = 3. Data were expressed as mean ± SD. Statistical significance is indicated as *P < 0.05 vs. the sham + vehicle groups, ^#^*P* < 0.05 vs. the LPS + vehicle groups.

More evidence supports that the activation of Nlrp3 is associated with various inflammatory diseases. In addition, the nuclear factor-κB (NF-κB) pathway is a primary regulator of inflammation, which enhances the expression of Nlrp3 in various cell types. Hence, the expression of phosphorylated-NF-κB (NF-κB/p65) was detected on protein level. Notably, the activation of *p*-NF-κB was downregulated by BBR treatment, which further suppressed the expression of Nlrp3 at both the transcriptional and posttranslational levels in both the *in vivo* and *in vitro* (Figures 4A–C). In addition, NF-κB is believed to be a transcriptional activator of the proinflammatory IL: IL-1β and IL-18. Moreover, LPS induced NF-κB nuclear translocation and increased the transcription of IL-1B and IL-18 (25). By consistent with the literature, we measured the mRNA expression level of *IL-1*β and *IL-18* in both lung tissue and HPMECs and found that the activation of pro-inflammation was inhibited by BBR treatment compared to vehicle treatment in the LPS-induced group (Figures 4D,E). Therefore, these results demonstrated that BBR restrained the expression of inflammatory cytokines by downregulating the activation of the NF-κB/p65-Nlrp3 signaling pathway.

### Downregulation of Nlrp3 Attenuates the Expression of Inflammatory Cytokines

**Figure 5:**
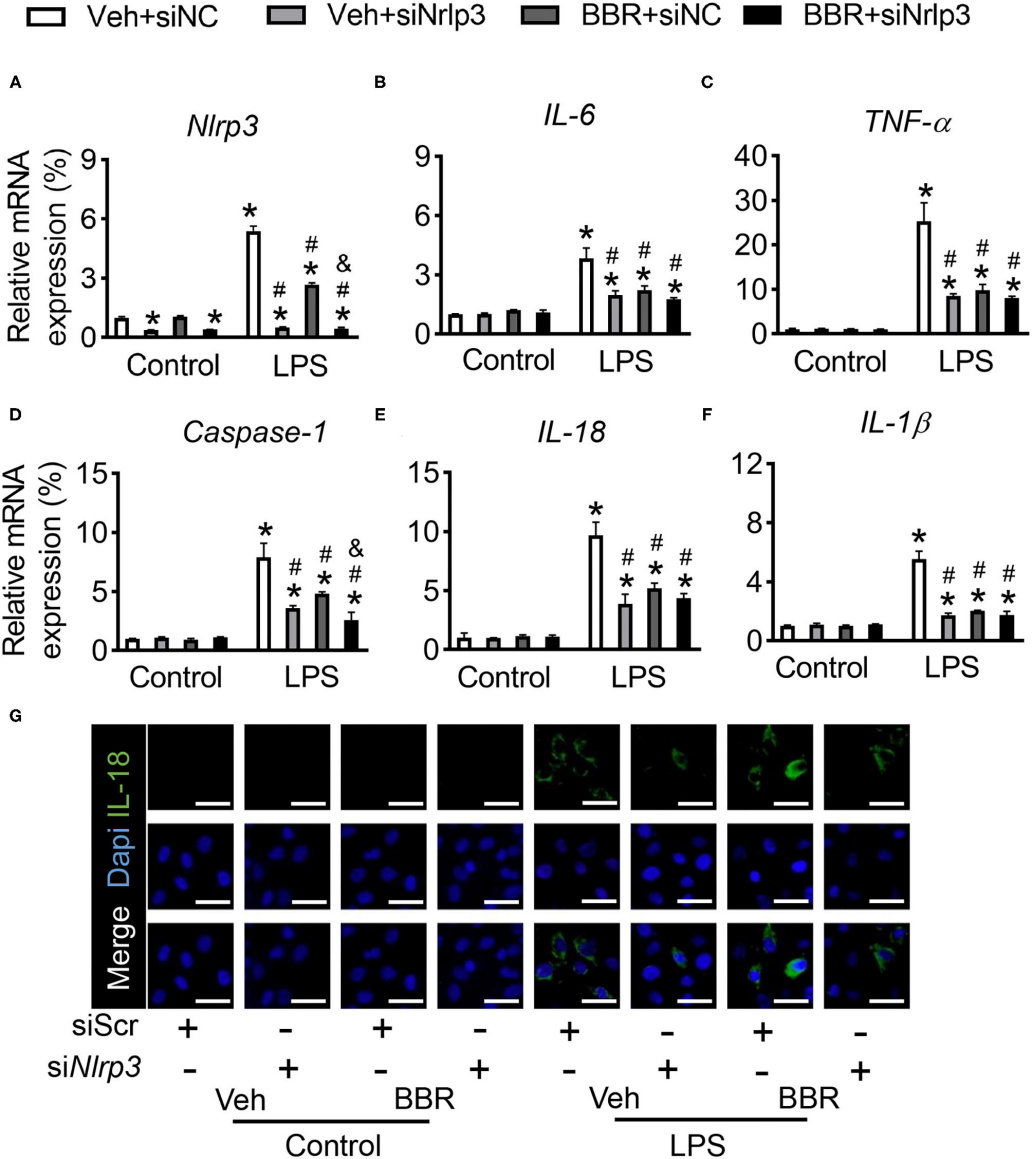
Downregulation of Nlrp3 repressed the treatment role of BBR in HPMECs induced with LPS. HPMECs were transfected with Nlrp3 siRNA and stimulated with LPS. **(A)** The mRNA expression of *Nlrp3*. **(B,C)** The mRNA expression of *IL-6* and *TNF-*α. **(D)** The mRNA expression of *Caspase-1 in vitro*. **(E,F)** The mRNA expression of *IL-1*β and *IL-18*. **(G)** The immunofluorescence staining of IL-18 in HPMECs, scale bar = 50 μm. Data were expressed as mean ± SD, *n* = 3 each group. Statistical significance is indicated as *P < 0.05 vs. the sham + vehicle groups, ^#^*P* < 0.05 vs. the LPS + vehicle groups and &*P* < 0.05 vs. the LPS + BBR groups.

Although the productions of IL-1β and IL-18 were detected, whether the inflammatory cytokines were regulated by directly transcriptional Nlrp3 or by Nlrp3-induced Nlrp3 Inflammasome Complex or both two pathways are not clear. Hence, we used siRNA of Nlrp3 to study the underlying mechanism. First, we detected the efficiency of siNlrp3 and found that the expression of Nlrp3 was significantly decreased by siNlrp3 whether stimulated with LPS or not (Figure 5A). In addition, compared with control siRNA-incubated HPMECs, the levels of inflammatory factors of *IL-6* and *TNF-*α were sharply repressed with siNlrp3 and BBR also restrained the LPS-stimulated enhancement in cytokines production (Figures 5B,C). As published assays revealed, the activation of inflammasome subsequently promoted the production of Caspase-1, which regulated the downstream genes of IL-1β and IL-18. Moreover, the mRNA expression level of *Caspase-1* was examined and found that siNlrp3 and BBR both significantly attenuated the expression after LPS treatment (Figure 5D). Consistently, the inflammatory factors of *IL-1*β and *IL-18* were also downregulated by siNlrp3 and BBR (Figures 5E,F). Further, the fluorescence staining of IL-18 demonstrated that the expression of IL-18 was obviously increased in the LPS-stimulated groups compared with the control groups, and this tendency was substantially reduced by treatment with BBR or siNlrp3 (Figure 5G). In general, these results demonstrated that BBR also could suppress the expression of inflammatory cytokines *via* attenuating the expression of Nlrp3.

### Berberine Is Able to Assemble Conformation With Nlrp3

**Figure 6:**
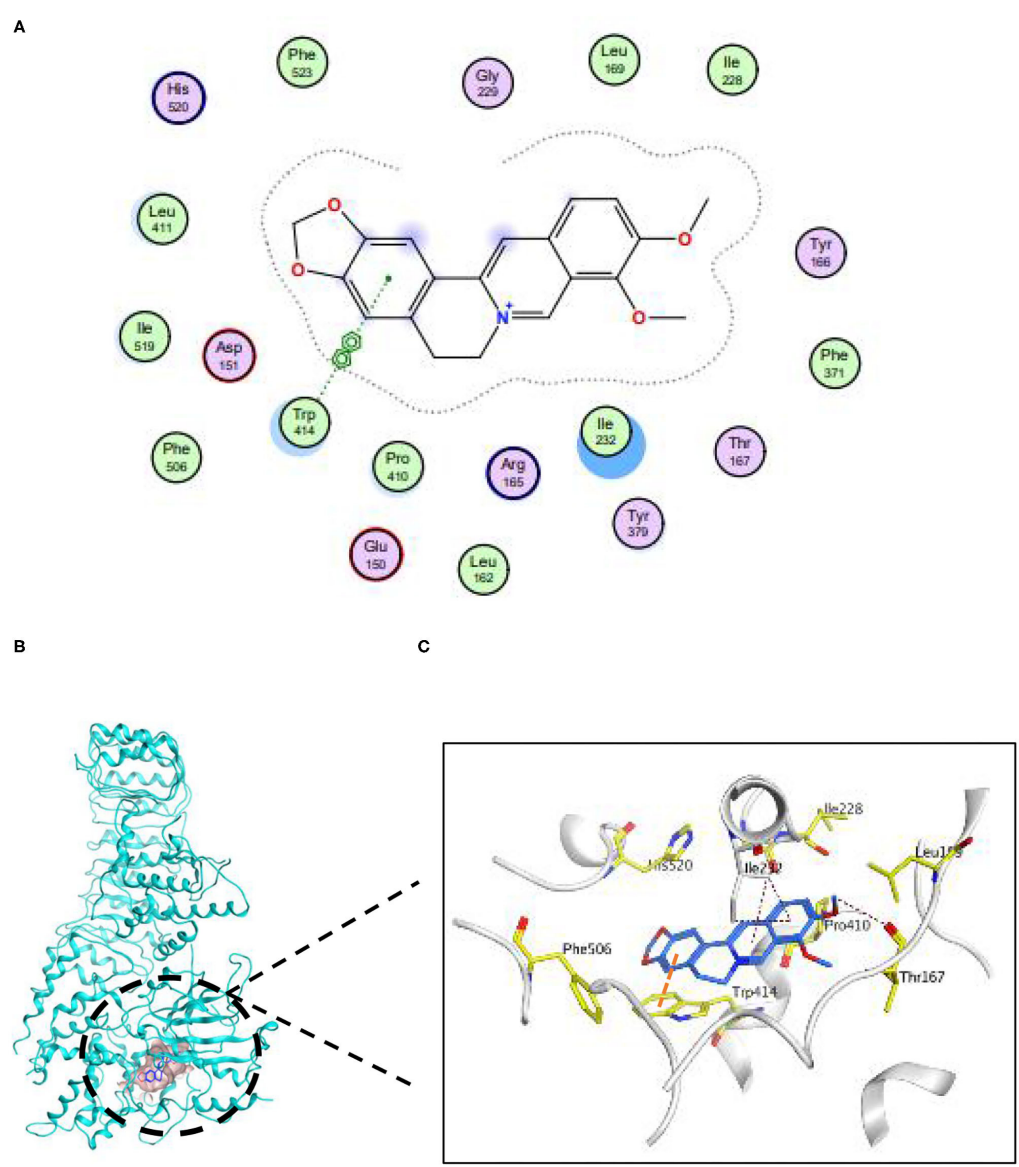
Berberine was capable of establishing conformation with Nlrp3. **(A)** The 2D binding pattern of BBR and Nlrp3. **(B)** The binding model of BBR on molecular surface of Nlrp3. The molecular surface of Nlrp3 was colored in pale blue and BBR was colored in cyan. **(C)** The 3D binding mode of BBR and Nlrp3.

To explore whether BBR directly combined with Nlrp3, we performed a molecular docking. The docking score of BBR with Nlrp3 was −7.27 (kcal/mol). The combination model of BBR with protein Nlrp3 was exhibited in Figure 6. The three pi-H conjugation interactions between BBR and Nlrp3 protein established a steric complementarity bridge. One oxygen atom of Thr167 was found as the acceptor of the hydrogen bond and built a hydrogen bond with a hydrogen atom of BBR. In addition, this binding site (Trp414) of the benzene ring forms pi–pi conjugation interaction with one benzene ring of BBR. Taken together, these interactions primarily caused potential binding energy between BBR with protein Nlrp3.

### Nlrp3 Deficiency Abrogates the Protective Function of BBR in ALI Mice

**Figure 7:**
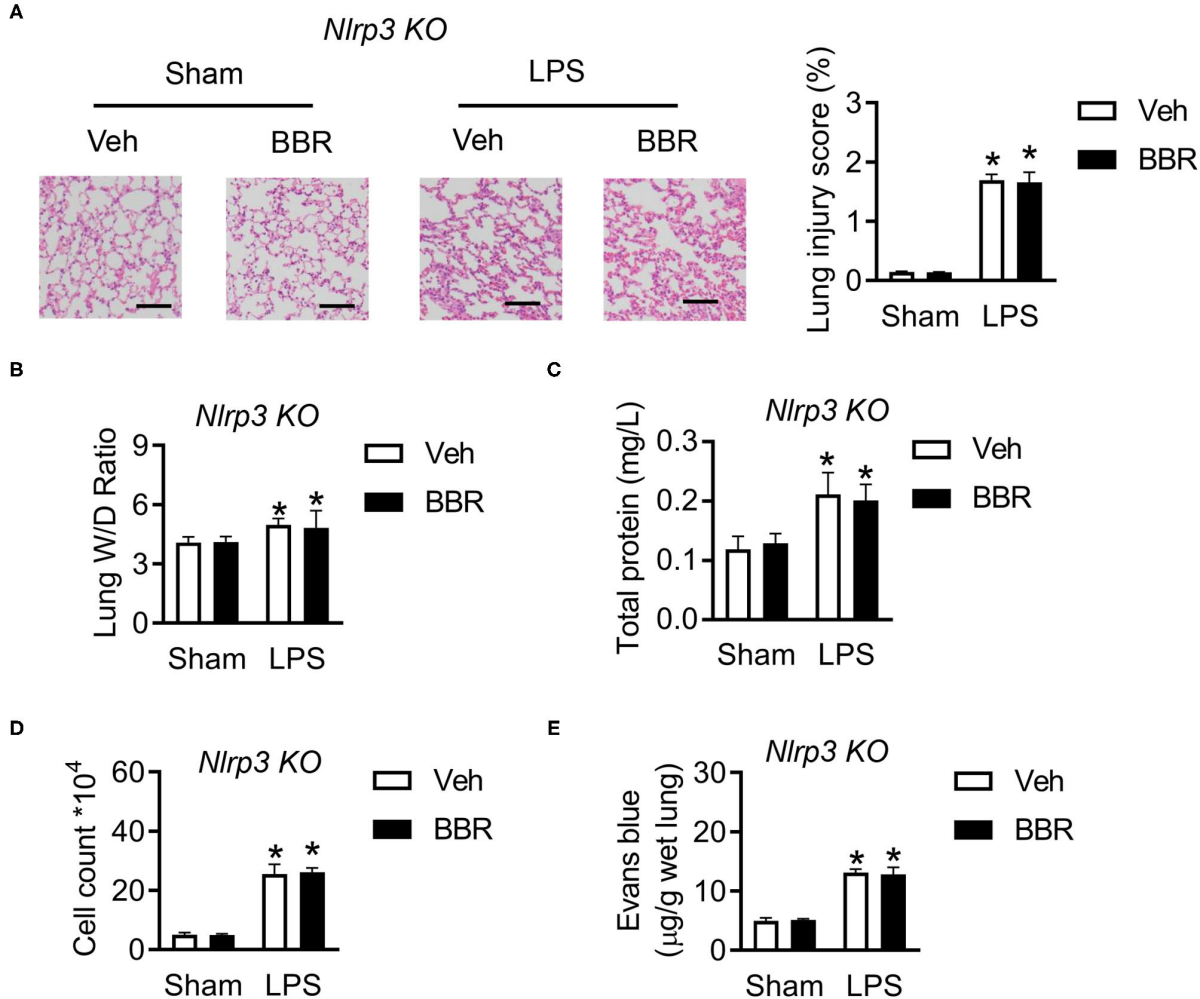
Deletion of Nlrp3 in mice abolished the protective role of BBR in ALI. **(A)** The H&E staining and lung injury score in pulmonary section, *n* = 3, scale bar = 100 μm. **(B)** The ratio of wet lung to dry lung, *n* = 8. **(C,D)** The detection of total protein and cell counts in BALF, *n* = 8. **(E)** The concentration of Evans blue in lung tissue, *n* = 8. Data were expressed as mean ± SD. Statistical significance is indicated as *P < 0.05 vs. the sham + vehicle groups. ALI, acute injury lung; BALF, bronchoalveolar lavage fluid.

The above results demonstrated that the protective function of BBR is dependent on the expression of Nlrp3, whether regulating the expression of phosphorylated-NF-κB or directly interacting with Nlrp3 protein. Hence, we established Nlrp3 knockout mice to perform further analysis. The Nlrp3 deficiency mice were injected LPS to produce ALI in a similar manner in wild-type mice. Several phenotypes were performed to verify the conjecture. The histological analysis demonstrated that Nlrp3 deficient mice in groups upon LPS stimulation exhibited approximate pulmonary injury, including interstitial edema, incomplete morphological structure of pulmonary, infiltration of inflammatory cells, and death of pulmonary endothelial cells, but BBR lost the protective role (Figure 7A). Additionally, the measure of permeability, the ratio of lung wet to dry weight, contents of total protein, cell count in BALF, and the leaking level of Evans Blue, all showed that BBR was not able to play a protective role in ALI based on the deficiency of Nlrp3 (Figures 7B–E). These results suggested that BBR protected ALI mice from numerous inflammation depends on Nlrp3.

## Discussion

This study has demonstrated that LPS stimulated excessive inflammatory cytokines and these promoted a series of inflammatory responses, which could be restrained by traditional Chinese medicine, named BBR. In addition, treatment with BBR suppressed pulmonary inflammatory factors and barrier dysfunction in the ALI mice model. Furthermore, the one underlying mechanism of BBR may be associated with the reduction of inflammatory cytokines *via* phosphorylated-NF-κB/Nlrp3 signal pathway, which protected the permeability of HPMECs and suppressed the expression of IL-1β and IL-18. Moreover, molecular docking demonstrated that BBR could be direct inhibition of the Nlrp3 protein, which regulated the Nlrp3/inflammasome signal pathway and attenuated the expression of IL-1β and IL-18. In general, both two signal pathways also regulated the key target of Nlrp3, and Nlrp3 deficient mice demonstrated that the protective function of BBR was depended on Nlrp3. Besides these, BBR can be inhibition of Nlrp3 and plays a therapeutic drug in ALI.

Berberine plays an important role in inhibiting inflammation *via* regulating the NF-κB signal pathway. BBR protects the brain against ischemia-reperfusion injury through inhibiting NF-κB nuclear translocation (26). Furthermore, the subarachnoid hemorrhage injury could be improved by suppressing the high mobility group box 1 (HMGB1)/NF-κB pathway (27). Moreover, BBR represses LPS-stimulated inflammatory factors in murine macrophage (RAW264.7 cells) by downregulating the Sirt1/NF-κB pathway (28). However, our results demonstrated that BBR could attenuate LPS-induced inflammation *via* restraining NF-κB/Nlrp3 signaling pathway.

Berberine could be direct inhibition of Nlrp3. A series of assays have demonstrated that BBR plays an anti-inflammation role and is associated with Nlrp3. The activation of mitophagy and the reduction of mitochondrial ROS restrained by BBR represses influenza virus-induced Nlrp3 inflammasome to improve lung injury (29). In addition, BBR could specifically interrupt the interaction of Nek7-Nlrp3 and subsequently decrease the expression of IL-1β (30). However, this study reveals that BBR cloud directly interacts with Nlrp3 protein to suppress the expression of Nlrp3 and successively inhibit the activation of the inflammasome signal pathway.

**Figure 8:**
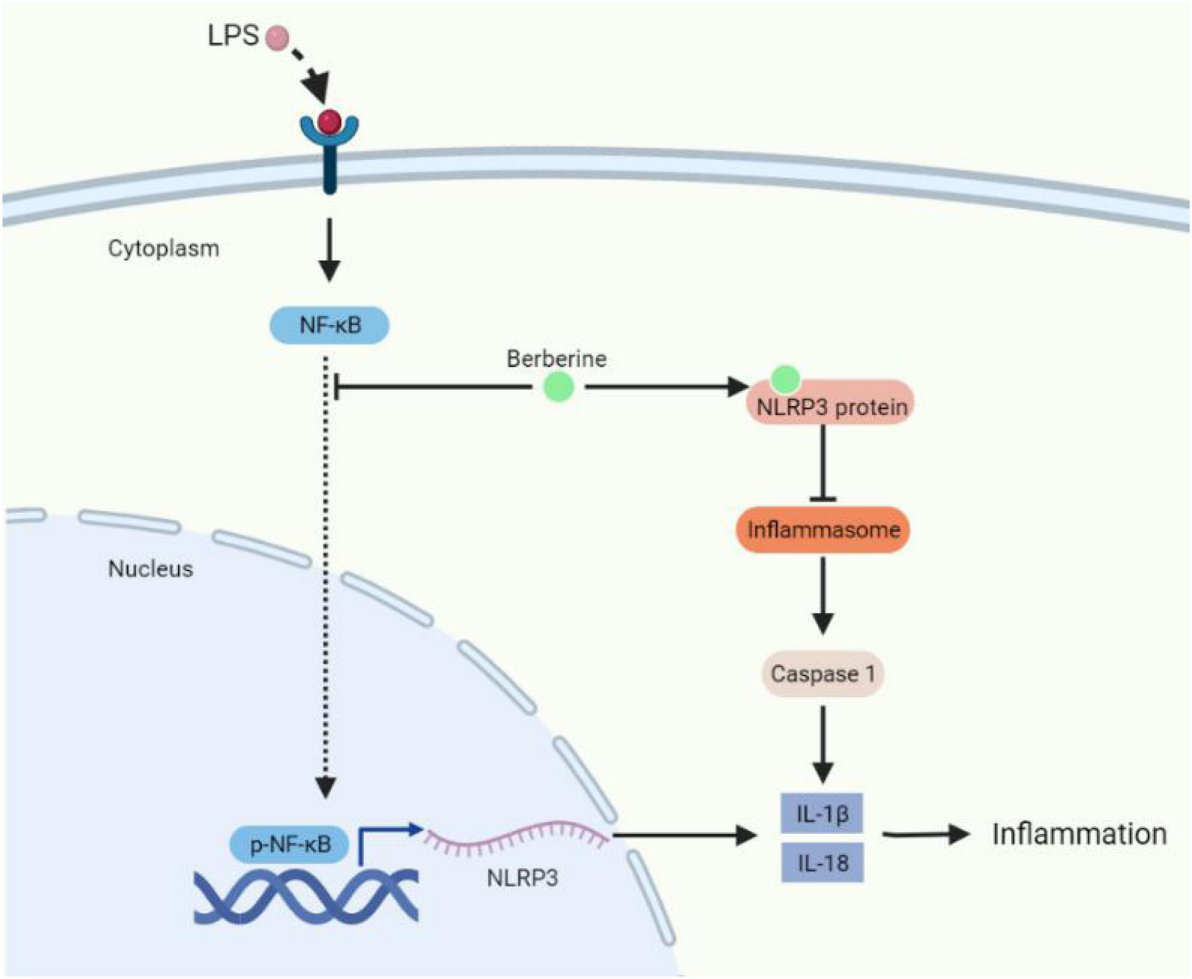
The molecular mechanism of berberine protecting lung injury under inflammatory conditions.

In general, we have researched the protective function of BBR on LPS-stimulated inflammation in animal and cell models. The underlying mechanisms may involve improving the permeability of endothelial cells, inhibiting inflammatory cytokines *via* NF-κB/Nlrp3 signaling pathway, and directly blocking Nlrp3 protein (Figure 8). These results provide an insight strategy of BBR as a potential therapeutics for ALI.

## Data Availability Statement

The raw data supporting the conclusions of this article will be made available by the authors, without undue reservation.

## Ethics Statement

The animal study was reviewed and approved by the Animal Experimental Ethics Committee of Guizhou Medical University (No. 2101452).

## Author Contributions

YH and XB: conceptualization, formal analysis, and investigation. JC and YH: methodology, writing—original draft preparation, and funding acquisition. YH: writing—review and editing. All authors contributed to the article and approved the submitted version.

## Funding

This study was supported by the Science and Technology Plan Project of Guizhou Province [qian ke he ji chu, (2020)1Y381], the Cultivation Plan of the NSFC (National Natural Science Foundation of China) of Guizhou Medical University (19NSP056), the Science and Technology Foundation of Health Commission of Guizhou Province (gzwkj2022-221), and the Administration of Traditional Chinese Medicine of Guizhou Province (QZYY-2018-130).

## Conflict of Interest

The authors declare that the research was conducted in the absence of any commercial or financial relationships that could be construed as a potential conflict of interest.

## Publisher's Note

All claims expressed in this article are solely those of the authors and do not necessarily represent those of their affiliated organizations, or those of the publisher, the editors and the reviewers. Any product that may be evaluated in this article, or claim that may be made by its manufacturer, is not guaranteed or endorsed by the publisher.
